# Supplementary material for: NEAT1 Long Isoform Is Highly Expressed in Chronic Lymphocytic Leukemia Irrespectively of Cytogenetic Groups or Clinical Outcome
Source: Noncoding RNA. 2020 Mar 13;6(1):11. doi: 10.3390/ncrna6010011 (PMC7151605; doi:10.3390/ncrna6010011)
Supplement: Supplementary file 1 [file ncrna-06-00011-s001.pdf]

Supplementary Material

NEAT1 long isoform is highly expressed in chronic lymphocytic leukemia irrespectively of cytogenetic groups or clinical outcome

Domenica Ronchetti,[1,2] Vanessa Favasuli,[1] Paola Monti,[3] Giovanna Cutrona,[4] Sonia Fabris,[2] Ilaria Silvestris,[1] Luca Agnelli,[1] Monica Colombo,[4] Paola Menichini,[3] Serena Matis,[4] Massimo Gentile,[5] Ramil Nurtdinov,[6] *Roderic Guigó,*[6] **Luca Baldini,[1,2] Gilberto Fronza,[3] Manlio Ferrarini,[7] Fortunato Morabito,[8,9] Antonino Neri,[1,2*] and Elisa Taiana.[1,2]**

[1] Department of Oncology and Hemato-oncology, University of Milan, 20122 Milan, Italy; domenica.ronchetti@unimi.it, ilaria.silvestris@unimi.it, vanessa.favasuli@unimi.it, luca.agnelli@unimi.it, luca.baldini@unimi.it, antonino.neri@unimi.it, elisa.taiana@.unimi.it

[2] Hematology, Fondazione Cà Granda IRCCS Policlinico, 20122 Milan, Italy; sonia.fabris1969@gmail.com

[3] Mutagenesis and Cancer Prevention Unit, IRCCS Ospedale Policlinico San Martino, 16132 Genova, Italy; paola.monti@hsanmartino.it, paola.menichini@hsanmartino.it, gilberto.fronza@hsanmartino.it

[4] Molecular Pathology Unit, IRCCS Ospedale Policlinico San Martino, 16132 Genova, Italy; giovanna.cutrona@gmail.com, monica.colombo@hsanmartino.it, serena.matis@hsanmartino.it

[5] Hematology Unit, Department of Onco-Hematology A.O. of Cosenza, 87100 Cosenza, Italy; massim.gentile@tiscali.it

[6] Centre for Genomic Regulation (CRG), The Barcelona Institute of Science and Technology, Dr. Aiguader 88, 08003 Barcelona, Catalonia, Spain; ramil.nurtdinov@crg.eu, roderic.guigo@crg.eu

[7] Department of Experimental Medicine, University of Genoa, 16126 Genoa, Italy; ferrarini.manlio@gmail.com

[8] Unità di Ricerca Biotecnologica, Azienda Sanitaria Provinciale di Cosenza, 87051 Aprigliano (CS), Italy; f.morabito53@gmail.com

[9] Department of Hematology and Bone Marrow Transplant Unit, 97300 Augusta Victoria Hospital, Jerusalem, Israel

* Correspondence: antonino.neri@unimi.it; Tel.: +39 02-50320420

Received: 21 January 2020; Accepted: 8 March 2020; Published: date

Supplementary Table S1. Molecular features of the four CLL patients showing the concomitant presence of del17p and TP53 mutation.

ID	cDNA_variant	Protein_variant	IGHV mutational status	TP53 VAF %	del(17)	Total NEAT1 expression (ΔCt)	NEAT1_2 expression (ΔCt)
DS0264	c.622_623insAATTTGGATG	p.Asp208Glufs	UM	95.1	+	-0.010	2.281
CR0203	c.772G>T	p.Glu258*	UM	97.7	+	1.934	3.374
FD0404	c.824G>A	p.Cys275Tyr	UM	63.6	+	1.929	2.883
DA0094	c.833C>G	p.Pro278Arg	UM	98.3	+	0.417	2.180

fs: frameshift

*stop codon

UM: unmutated

VAF: variant allele frequency

Supplementary Figure S1

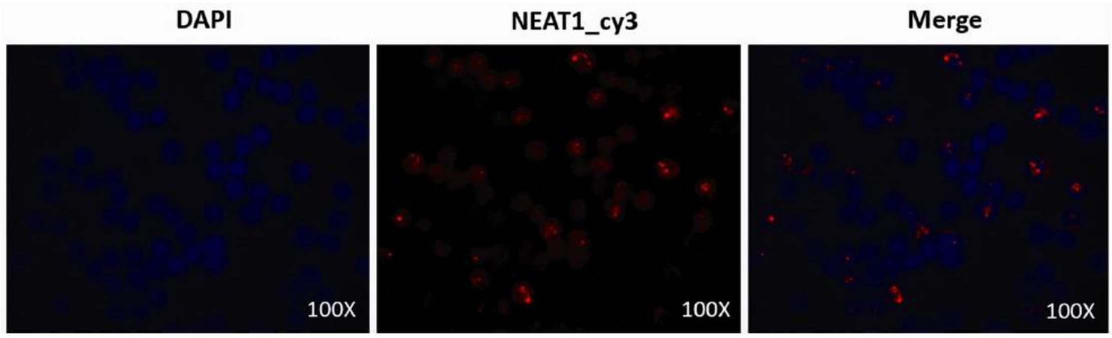

Supplementary Figure S1. NEAT1 RNA FISH detection. Example of NEAT1 aggregates in the nucleus of peripheral mononuclear cells purified from a CLL patient by Ficoll-Hypaque (Lonza Group, Basel, Switzerland) density gradient sedimentation; CD5-CD19 percentage was >70%. We used the Stellaris RNA FISH kit (Biosearch Technologies), according to the manufacturer’s instructions. For NEAT1 detection, we took advantage of a commercial set of Quasar® 570-labeled oligos (Stellaris, Biosearch Technologies) able to bind the 5′ end of NEAT1 transcript.

Supplementary Figure S2

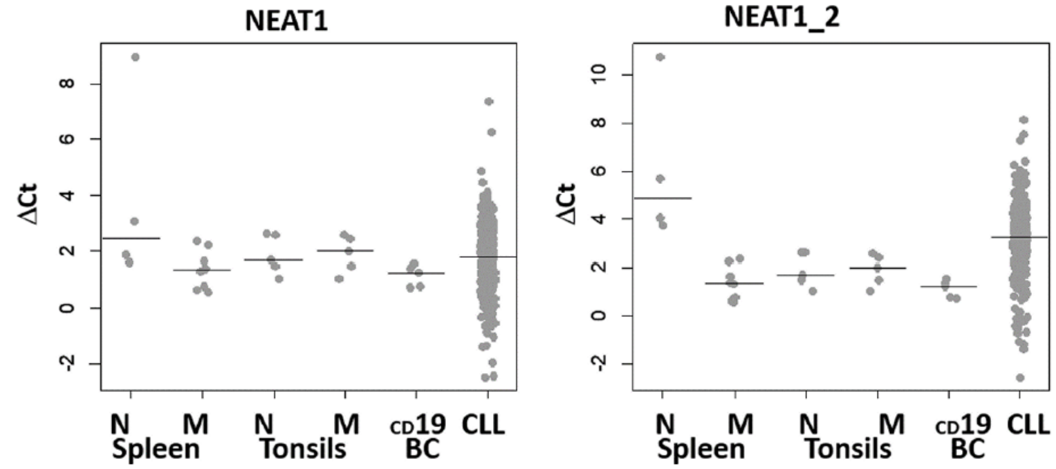

Supplementary Figure S2. NEAT1 expression level in CLL. Stripchart of NEAT1 and NEAT1_2 expression levels evaluated by qRT-PCR in CLL and in normal samples, including naïve cells (N) purified from spleen (4 samples) and tonsils (5 samples), memory cells (M) from spleen (8 samples) and tonsils (5 samples), and CD19 BC (5 samples). Expression data are reported as ΔCt referred to GAPDH housekeeping gene. No significant differences were found at $p < 0.01$.

Supplementary Figure S3

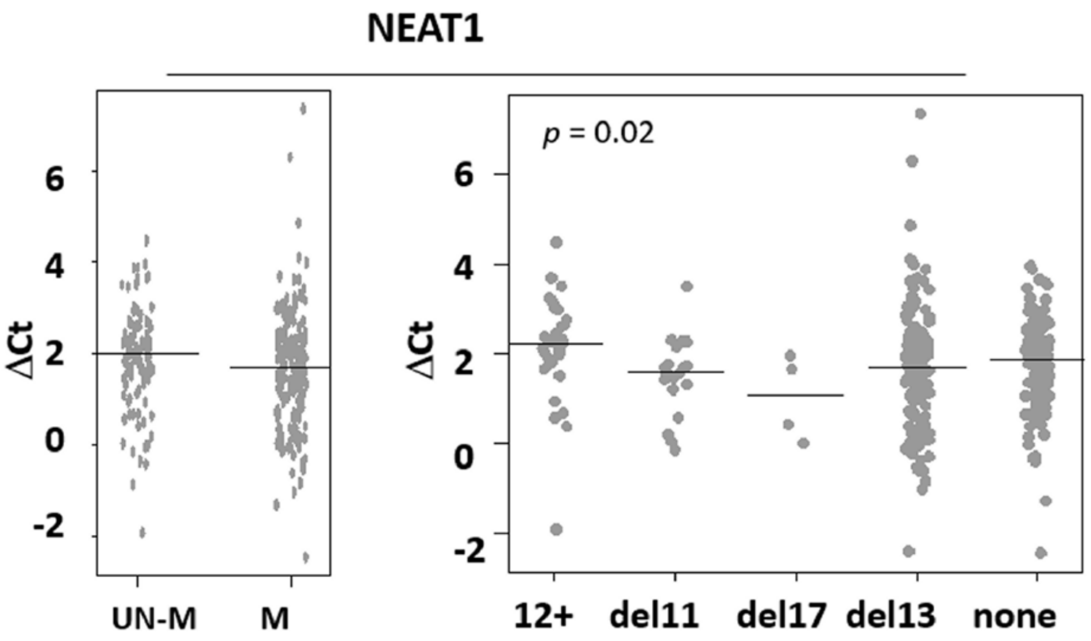

Supplementary Figure S3. Stripchart of NEAT1 expression in CLL subgroup defined by mutational IGVH status or the presence of the main chromosomal abnormalities detected by FISH. Expression data are reported as ◎Ct referred to GAPDH housekeeping gene. No significant differences were found at $p < 0.01$.

Supplementary figure S4

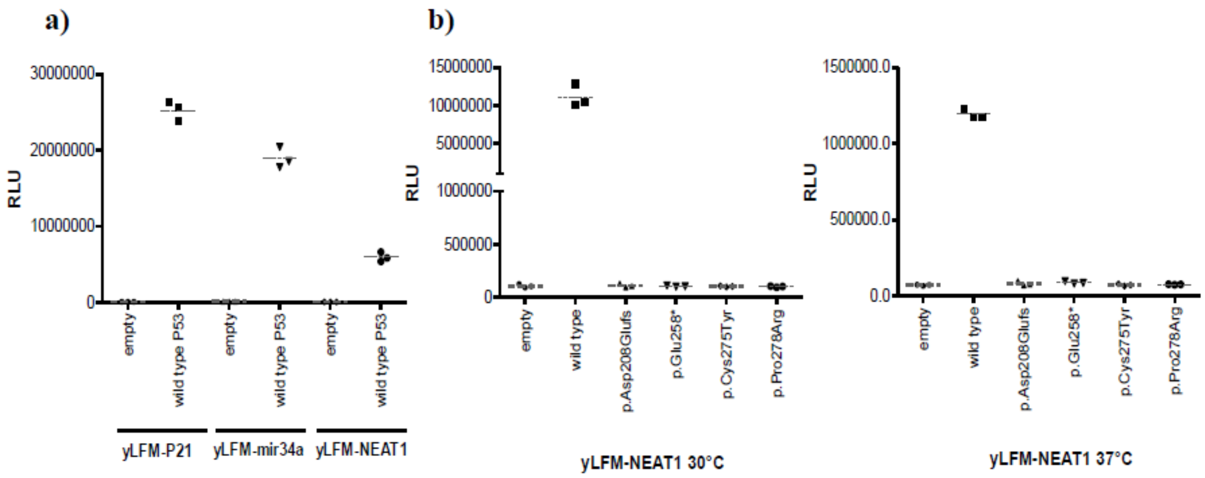

Supplementary figure S4. **yeast-based P53 functional assay (a)** Transactivation ability of wild type P53 protein by using a yeast-based functional assay. The average relative light units (RLU) of three biological replicates are shown. Yeast cells have been grown for 8 hours in the presence of galactose (0,128%) to induce P53 expression. The P53 RE from NEAT1 promoter is transactivated by wild type P53 protein, although more weakly than P53 REs from P21 and mir34a promoters. **(b)** Transactivation ability of P53 mutants (harboring the TP53 mutations identified in our cohort of CLL patients) in yLFM-NEAT1 reporter strain. The average RLU and standard deviations measured are shown, as in panel (a) by growing yeast cells at 30°C and 37°C. In both tested conditions, all P53 mutants lose the transactivation ability with respect to wild type P53.
